# HIF-1 and NRF2; Key Molecules for Malignant Phenotypes of Pancreatic Cancer

**DOI:** 10.3390/cancers14020411

**Published:** 2022-01-14

**Authors:** Shin Hamada, Ryotaro Matsumoto, Atsushi Masamune

**Affiliations:** Division of Gastroenterology, Tohoku University Graduate School of Medicine, Sendai 980-8574, Japan; hamadas@med.tohoku.ac.jp (S.H.); rmat44@gmail.com (R.M.)

**Keywords:** HIF-1, KEAP1, NRF2, hypoxia, microenvironment, oxidative stress, pancreatic stellate cells

## Abstract

**Simple Summary:**

Pancreatic cancer progression involves interactions between cancer cells and stromal cells in harsh tumor microenvironments, which are characterized by hypoxia, few nutrients, and oxidative stress. Clinically, cancer cells overcome therapeutic interventions, such as chemotherapy and radiotherapy, to continue to survive. Activation of the adaptation mechanism is required for cancer cell survival under these conditions, and it also contributes to the acquisition of the malignant phenotype. Stromal cells, especially pancreatic stellate cells, play a critical role in the formation of a cancer-promoting microenvironment. We here review the roles of key molecules, hypoxia inducible factor-1 and KEAP1-NRF2, in stress response mechanisms for the adaptation to hypoxia and oxidative stress in pancreatic cancer cells and stellate cells. Various cancer-promoting properties associated with these molecules have been identified, and they might serve as novel therapeutic targets in the future.

**Abstract:**

Pancreatic cancer is intractable due to early progression and resistance to conventional therapy. Dense fibrotic stroma, known as desmoplasia, is a characteristic feature of pancreatic cancer, and develops through the interactions between pancreatic cancer cells and stromal cells, including pancreatic stellate cells. Dense stroma forms harsh tumor microenvironments characterized by hypoxia, few nutrients, and oxidative stress. Pancreatic cancer cells as well as pancreatic stellate cells survive in the harsh microenvironments through the altered expression of signaling molecules, transporters, and metabolic enzymes governed by various stress response mechanisms. Hypoxia inducible factor-1 and KEAP1-NRF2, stress response mechanisms for hypoxia and oxidative stress, respectively, contribute to the aggressive behaviors of pancreatic cancer. These key molecules for stress response mechanisms are activated, both in pancreatic cancer cells and in pancreatic stellate cells. Both factors are involved in the mutual activation of cancer cells and stellate cells, by inducing cancer-promoting signals and their mediators. Therapeutic interventions targeting these pathways are promising approaches for novel therapies. In this review, we summarize the roles of stress response mechanisms, focusing on hypoxia inducible factor-1 and KEAP1-NRF2, in pancreatic cancer. In addition, we discuss the potential of targeting these molecules for the treatment of pancreatic cancer.

## 1. Introduction

The prognosis of pancreatic cancer is poor, despite improvements in therapeutic options, and incidences are increasing [1]. Patients with pancreatic cancer are generally diagnosed at an advanced, unresectable stage. In these cases, the median survival time is less than 12 months [2]. Clinically detectable pancreatic cancer develops through the long-lasting interactions between cancer cells and host cells, which persists for more than 10 years [3]. Conventional chemotherapeutic agents, such as gemcitabine, induce resistance in cancer cells through continuous administration [4]. These therapeutic interventions act as constant stressors that lead to the selection of malignant cancer cells, resulting in therapeutic resistance and poor prognosis. Perpetuated adaptation to a harsh microenvironment and therapeutic intervention itself forms a feedforward loop for the malignant phenotype of cancer cells. Pancreatic cancer cells can survive in a harsh microenvironment, characterized by poor nutrients, low oxygen, and/or immunosuppression, for a long time. In this review, we summarize the roles of stress response mechanisms in pancreatic cancer. In addition, we discuss the potential of targeting these stress response mechanisms for the treatment of pancreatic cancer.

## 2. Dense Fibrotic Stroma in Pancreatic Cancer

Dense fibrotic stroma, known as desmoplasia, is a characteristic feature of pancreatic cancer [5]. Desmoplasia develops through the interactions between pancreatic cancer cells and stromal cells, including pancreatic stellate cells (PSCs) [6]. PSCs play critical roles in the development of pancreatic fibrosis by producing extracellular matrix (ECM) proteins, such as collagen and fibronectin [6]. Interaction between cancer cells and PSCs enhances the malignant potential of pancreatic cancer cells, such as epithelial-mesenchymal transition (EMT) [7] and cancer stem cell-related marker expression [8]. Soluble factors from PSCs activate multiple signaling pathways, such as the signal transducer and activator of transcription 3, Akt, p38 mitogen-activated protein kinase, and extracellular signal-regulated kinase in pancreatic cancer cells [9]. A previous study tested the sonic hedgehog inhibitor IPI-926 in a pancreatic cancer mouse model, which improved the response to chemotherapy for a short period [10]. However, longer administration or deletion of PSCs paradoxically promoted cancer progression, resulting in the shorter survival of model mice [11]. Another study identified a cancer progression-suppressing population in PSCs expressing the undifferentiated mesenchymal stem cell marker meflin [12]. Deletion of this glycosylphosphatidylinositol-anchored protein promoted pancreatic cancer progression, suggesting that a simple deletion strategy for PSCs is insufficient.

Due to the limited formation of an efficient vascular network, dense stroma forms harsh tumor microenvironments for pancreatic cancer cells and PSCs, characterized by hypoxia, few nutrients, oxidative stress, and acidic extracellular pH [13,14]. Furthermore, several types of cells, such as myeloid-derived suppressor cells, tumor-associated macrophages, regulatory T cells, cancer-associated fibroblasts, and mast cells, act for immunosuppression in the tumor microenvironment [15]. Increased reactive oxygen species (ROS) production from myeloid-derived suppressor cells suppressed T cell functions, leading to immunosuppression in a wide variety of cancer models, such as mammary carcinoma, colon carcinoma, and lung carcinoma [16]. ROS also induced M2 polarization of tumor-associated macrophages, resulting in immunosuppression in lung cancer models [17]. ECM proteins such as fibronectin and laminin stimulated ROS production in cancer cells, leading to an increase of oxidative stress [18]. Different concentrations of ROS exert biphasic biological effects on cancer progression [19,20]. Activation of multiple signaling pathways by adequate levels of ROS promotes cancer progression via EMT induction and growth promotion. However, a higher level of ROS triggers cell death via apoptosis, necrosis, and ferroptosis. Antitumor drugs such as paclitaxel and daunorubicin induced necrosis of cancer cells through the increased intracellular levels of ROS in pancreatic cancer [21]. This severe microenvironment gives continuous stresses to both pancreatic cancer cells and PSCs. Pancreatic cancer cells as well as PSCs survive in the harsh microenvironments through the altered expression of signaling molecules, transporters, and metabolic enzymes governed by various stress response mechanisms. Several stress response mechanisms enable adaptation to the microenvironment and cancer cell survival. Hypoxia and oxidative stress are two major stressors that directly lead to cell death. The adaptation mechanisms for these conditions have been studied, and the central regulators of hypoxia and oxidative stress responses have been identified: hypoxia-inducible factor-1 (HIF-1) and NRF2, respectively [22,23]. These mechanisms also yield growth advantages, metabolic reprogramming, and malignant phenotypes of cancer cells. Furthermore, these mechanisms play pivotal roles in PSCs, contributing to the formation of a cancer-promoting microenvironment. These complex interactions have been studied in the past decade, leading to the identification of significant contributions to pancreatic cancer progression and therapy resistance. 

## 3. Hypoxia-Inducible Factor 1: A Central Machinery for Hypoxia Response Mechanism

Desmoplasia physically hampers blood perfusion, resulting in low oxygen pressure within the pancreatic cancer microenvironment. Intraoperative measurements of pancreatic cancer oxygenation revealed significant hypoxia compared to adjacent normal pancreas [24]. Cellular adaptation to hypoxia has been well-studied, and the transcriptional factor HIF-1 has been identified as a central machinery. HIF-1 is a heterodimer composed of an α-subunit and a β-subunit. This heterodimer recognizes hypoxia-responsive elements of HIF-1 target genes and upregulates their expression in response to hypoxia [22]. The expression level of HIF-1β is stable; however, HIF-1α expression increases under hypoxic conditions. The low oxygen detection mechanism relies on the interplay between HIF-1α and proteasomal degradation. Under normoxia, Pro402 and Pro564 of HIF-1α are hydroxylated by prolyl hydroxylase, which facilitates ubiquitination by the von Hippel–Lindau (VHL) tumor suppressor protein. Hypoxic conditions attenuate hydroxylation of HIF-1α, leading to stabilization and nuclear translocation [22]. The major role of HIF-1 is induction of vascular endothelial growth factor (VEGF), a potent angiogenic factor [25]. Cancer cells proliferate by using this reaction to promote neovascularization, which aids in the dissemination of cancer cells. Following an increase in HIF-1, its target genes trigger metabolic reprogramming, such as increased glucose uptake, glycolysis, lactate production, and amino acid utilization. These effects are mediated by the upregulation of glucose transporters (GLUT1 and GLUT3), glycolytic enzymes (hexokinase, pyruvate kinase, lactate dehydrogenase, and others), and amino acid transporters in various types of cancer [26,27,28]. In addition to reactive activation of HIF-1, constitutive activation of HIF-1α by loss-of-function mutation of the *VHL* gene is the causative mutation of von Hippel–Lindau disease, a familial cancer syndrome characterized by renal cell carcinoma, pheochromocytoma, and hemangioblastoma (central nervous system and retina) [29]. 

## 4. Effects of Hypoxia on Pancreatic Cancer Cells

Glucose-deprived hypoxic conditions induce glucose transporters and the angiogenic factor VEGF in pancreatic cancer cells [30]. Another study that identified transcriptional induction of hepatocyte growth factor activator by HIF-1 led to the activation of the hepatocyte growth factor/c-Met signaling pathway and invasiveness of pancreatic cancer cells [31]. The urokinase-type plasminogen activator receptor (uPAR) plays a pivotal role in angioinvasion to establish distant metastasis. The promoter region of the *uPAR* gene contains a HIF-1 binding site, and hypoxic treatment upregulated uPAR expression and the invasive capacity of pancreatic cancer cells [32]. HIF-1 activation also induced metabolic reprogramming in pancreatic cancer cells. Hypoxic treatment of human pancreatic cancer cell lines increased prolyl 4-hydroxylase subunit alpha 1 in a HIF-1-dependent manner [33]. The prolyl 4-hydroxylase subunit alpha 1 stabilized HIF-1α, acting as a positive feedback loop. The prolyl 4-hydroxylase subunit alpha 1 contributed to glycolytic activity, cellular proliferation, and chemoresistance. Pyruvate dehydrogenase converts pyruvate to acetyl-CoA, which is essential for mitochondrial oxidative phosphorylation [34]. Hypoxia-induced repression of pyruvate dehydrogenase activity was mediated by pyruvate dehydrogenase kinase 1 in pancreatic cancer cells [35]. This metabolic reprogramming led to glycolysis dependence, and knockdown of HIF-1α or pyruvate dehydrogenase kinase 1 restored pyruvate dehydrogenase activity and repressed xenografted tumor growth in immunodeficient mice, suggesting a contribution of pyruvate dehydrogenase repression in pancreatic cancer progression [35]. Hypoxia-induced HIF-1 activation affected the migratory ability of cancer cells. Treatment of human pancreatic cancer cells with hypoxia changed cellular morphology as spindle-like cells with less cell-to-cell adhesion, compatible to EMT [36]. Along with the HIF-1α accumulation, the EMT-inducing transcriptional factor TWIST expression was observed. Knockdown of HIF-1α led to the loss of TWIST induction and EMT induction by hypoxia. Hypoxia induced pro-fibrogenic factors such as connective tissue growth factor (CTGF) via HIF-1. CTGF plays a pivotal role in renal fibrosis and skin fibrosis [37,38]. CTGF protected pancreatic cancer cells from hypoxia-mediated apoptosis [39]. CTGF also contributed to gemcitabine-resistant phenotype in cancer cells [40]. Furthermore, HIF-1 mediated immune evasion and enhanced cancer stem cell properties and autophagy in pancreatic cancer cells [41]. 

**Figure 1:**
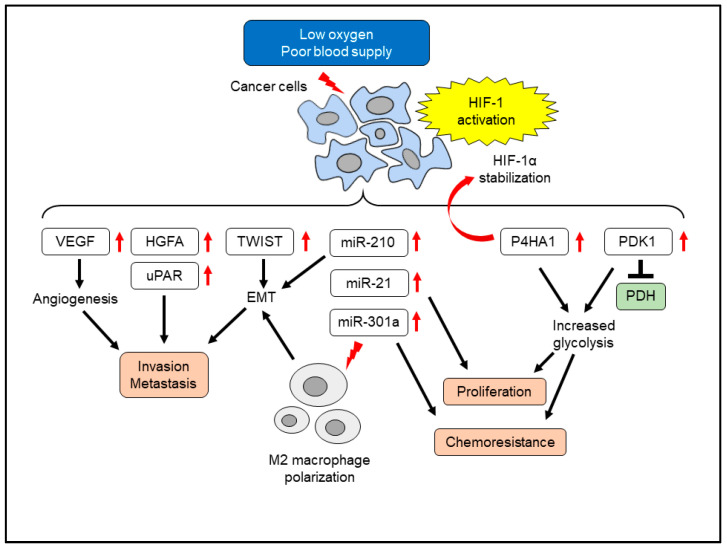
Effects of hypoxia in pancreatic cancer cells. EMT, epithelial-mesenchymal transition; HGFA, hepatocyte growth factor activator; HIF-1, hypoxia-inducible factor 1; P4HA1, prolyl 4-hydroxylase subunit alpha 1; PDH, pyruvate dehydrogenase; PDK1, pyruvate dehydrogenase kinase 1; uPAR, urokinase-type plasminogen activator receptor; VEGF, vascular endothelial growth factor.

In addition to these conventional growth factors and signaling molecules, hypoxia also affects the expression of microRNAs such as miR-21 and miR-210 [42,43]. MicroRNAs are single-stranded non-coding RNAs consisting of 21–24 nucleotides, which have various regulatory roles in cellular functions [44]. MiR-210 is referred to as “hypoxiamiR”, which is robustly induced by hypoxia in a wide variety of cells [43]. Elevated expression of miR-210 was associated with poor survival of patients with pancreatic cancer, suggesting its cancer-promoting role [45]. In addition to hypoxia, PSCs by themselves induced miR-210 expression in pancreatic cancer cells [46]. Inhibition of miR-210 in pancreatic cancer cells suppressed PSC-induced EMT, suggesting a role of miR-210 in the cancer-promoting interactions between PSCs and cancer cells. MiR-21 regulated migration, invasion, and chemoresistance in pancreatic cancer cells [47]. The cancer-promoting miR-21 expression was also increased by hypoxia in a HIF-1α-dependent manner [42]. MiR-21 overexpression promoted pancreatic cancer cell proliferation, even under hypoxia. Another hypoxia-inducible miRNA, miR-301a, induced gemcitabine resistance in human pancreatic cancer cells. Overexpression of miR-301a also induced gemcitabine resistance [48]. This microRNA directly targeted TAp63 and PTEN, which led to the resistance to gemcitabine. MiR-301a was also released from hypoxic cancer cells via a small extracellular vesicle called exosome. The exosomal miR-301a repressed PTEN in recipient macrophages, leading to M2 polarization in pancreatic cancer [49]. These M2 type macrophages promoted the EMT of cancer cells, contributing to the malignant phenotype. In summary, hypoxia affects multiple functions of cancer cells by a wide variety of mediators (Figure 1). 

## 5. Effects of Hypoxia on PSCs

**Figure 2:**
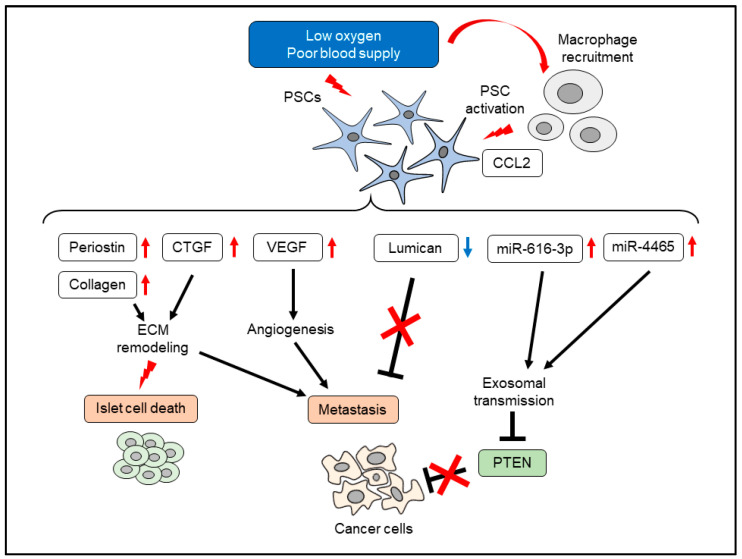
Effects of hypoxia in PSCs. CCL2, chemical chemokines 2; CTGF, connective tissue growth factor; VEGF, vascular endothelial growth factor.

Hypoxia also affects the cellular functions of PSCs. Hypoxia induced migration, type I collagen production, and VEGF production in PSCs [50]. Conditioned media of PSCs increased the tube formation on Matrigel in vitro and directed vessel formation in nude mice in vivo. [50]. ECM proteins, such as periostin, deposits around the capillaries of pancreatic cancer, and hypoxia increased periostin expression in PSCs [51]. Similarly, hypoxia-treated PSCs secreted significant amounts of CTGF, which promoted the invasive potential of pancreatic cancer cells [52]. Knockdown of CTGF by RNA interference blunted this effect. CTGF expression was observed in PSCs within surgically resected pancreatic cancer tissue, along with the marker of hypoxia, carbonic anhydrase 9 [52]. Hypoxia altered the ECM fiber organization produced by PSCs. A gelatin-based 3D matrix culture enabled the recapitulation of cell-free 3D matrices produced by PSCs. Hypoxia altered ECM fiber organization as a parallel pattern of fibronectin, which promoted the directional migration of pancreatic cancer cells [53]. Despite the cancer-promoting roles of PSCs, other types of cells, such as islet cells, are damaged by PSCs. A previous study showed that PSCs reduced insulin expression and induced β-cell apoptosis [54]. Diphenylene iodonium (DPI), an inhibitor of PSC activation, protected islet cells in WBN/Kob rats, an experimental model of chronic pancreatitis [54]. Hypoxia-activated PSCs increased β-cell death via elevated ROS production in PSCs [55]. Interestingly, hypoxia also affects the cancer-suppressing effects of PSCs. PSCs produce lumican, a small leucine-rich proteoglycan, which inhibited pancreatic cancer cell growth via EGFR reduction and reduction of Akt activity [56]. Expression of stromal lumican was correlated with reduced metastatic recurrence and longer survival [56]. Hypoxia repressed lumican production from PSCs through the increased autophagic flux supported by HIF-1α and activation of AMP-regulated protein kinase. Reduction of lumican production was reversed by autophagy inhibition [57]. The hypoxic microenvironment itself recruited macrophages by chemical chemokines 2 production, which activated PSCs [58]. Chemical chemokines 2 derived from hypoxic cancer cells recruited macrophages, and these macrophages increased αSMA expression in PSCs. Compared to cancer cells, hypoxia-regulated microRNAs are few in PSCs. Hypoxia increased miR-4465 and miR-616-3p in PSCs, leading to increased proliferation, migration, and invasion of pancreatic cancer cells via exosomal transmission [59]. These effects were mediated by PTEN reduction, which was a direct target of miR-4465 and miR-616-3p [59]. Taken together, responses to hypoxia in cancer cells and PSCs result in mutual activation, which further promotes a cancer-promoting microenvironment synergistically (Figure 2). 

## 6. KEAP1-NRF2 System: A Central Machinery for Oxidative Stress Response

**Figure 3:**
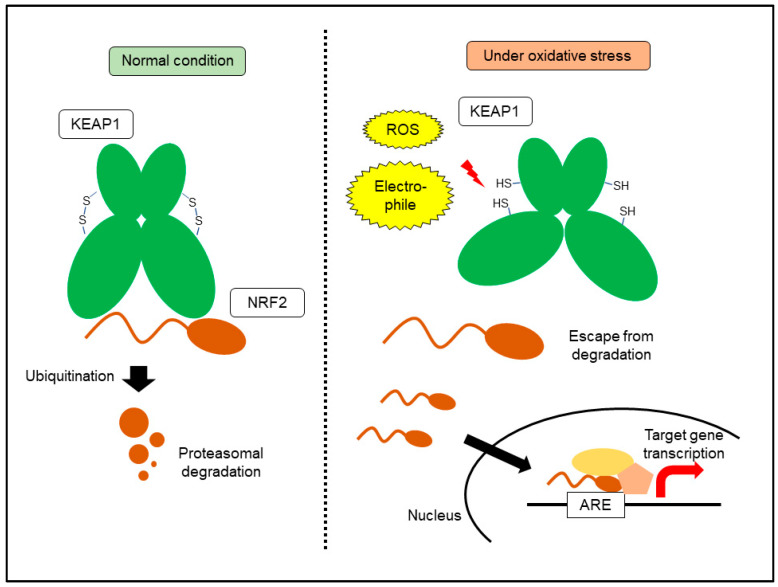
NRF2 activation by oxidative stress. ARE, antioxidant response element; ROS, reactive oxygen species.

Energy production by oxygen respiration coincides with the lethal damage caused by increased oxidative stress. Increased oxidative and electrophilic stressors need to be scavenged immediately to prevent cell death and organ damage. The KEAP1-NRF2 system is the central machinery that regulates oxidative stress responses [23,60]. NRF2 is a transcription factor that recognizes antioxidant response elements in its target genes. NRF2 binds to DNA as a heterodimer with small musculo-aponeurotic fibrosarcoma proteins. NRF2 interacts with cAMP responsive element binding protein and BRG1, a histone acetyltransferase and a component of the SWI/SNF chromatin remodeling complex, respectively, leading to an increase in target gene transcription [61]. Under normal conditions, NRF2 is degraded by proteasomes. KEAP1 forms a ubiquitin ligase complex, which binds to NRF2 to promote degradation by ubiquitination [23]. Increased ROS and electrophilic xenobiotics attack disulfide bonds within KEAP1, resulting in a conformational change that prevents binding to NRF2. NRF2 translocates into the nucleus and activates the transcription of target genes. KEAP1 is a thiol-based sensor molecule containing many cysteine residues. To date, three major cysteine residues of KEAP1 have been identified, which are critical for ubiquitin ligase complex activity. Interestingly, these cysteine residues are affected by distinct electrophiles [61]. A wide variety of transporters and metabolic enzymes are included in NRF2 target genes, leading to a reduction in oxidative stress. Detoxication/antioxidant enzymes include cysteine/glutamate transporter, glutathione peroxidase, glutathione peroxidase, glutathione S-transferase, heme oxygenase 1, Nqo1, and thioredoxin [60]. After the reduction of oxidative stress, the KEAP1 structure recovers and NRF2 is again degraded (Figure 3). Mutation-based activation of NRF2 exists in cancer cells, leading to constitutive activation [62]. Loss-of-function mutations in *KEAP1*, degradation-resistant *NRF2* mutations, and defective mutations of ubiquitin ligase *CUL3* have been reported in lung or esophageal cancer cells [62]. Epigenetic silencing of *KEAP1* and increased accumulation of competitive inhibitors of NRF2 binding, such as p62, also contribute to NRF2 activation in a wide variety of cancers. *KEAP1*- or *NRF2*-mutation-based constitutive activations have been reported in non-small cell lung cancer and esophageal cancer [63,64,65]. The existence of *KEAP1* or *NRF2* mutations in patients with non-small cell lung cancer was correlated with poor responses to chemotherapy, suggesting that NRF2 activation causes resistance to therapeutic intervention. Alternative activation of NRF2 via p62 has been reported in hepatocellular carcinoma (HCC). Accumulation of p62 was essential for NRF2 activation and *c-Myc* induction in HCC and contributed to the survival of HCC-initiating cells [66]. Another study reported that Ser351 phosphorylation of p62 further increased Nrf2 activation, leading to metabolic reprogramming and an increased malignant phenotype in a mouse model of HCC. In human HCC, Ser349-phosphorylated p62 (corresponding to mouse Ser351) accumulation was frequently observed in hepatitis C virus-positive patients with HCC [67]. In pancreatic cancer, overexpressed ataxia-telangiectasia group D-associated gene products bind to KEAP1, leading to NRF2 activation [68]. 

## 7. Effects of NRF2 Activation in Pancreatic Cancer Cells

**Figure 4:**
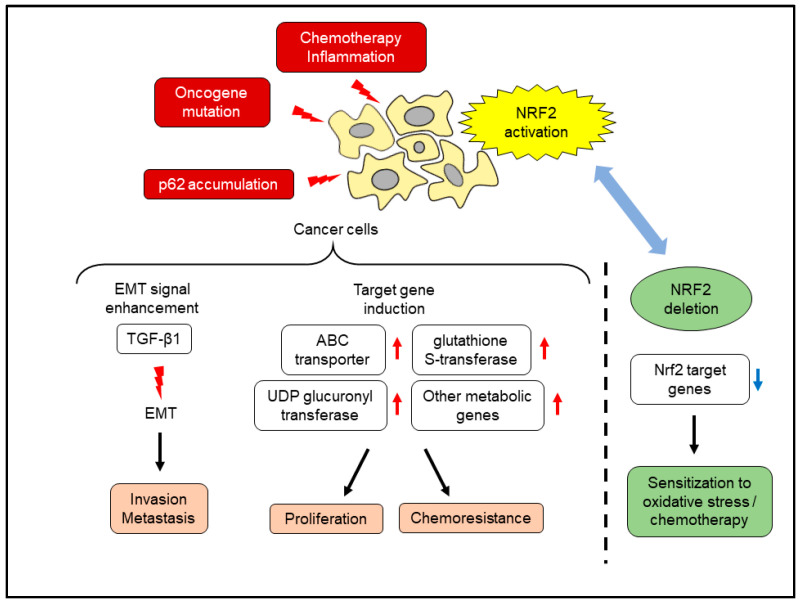
Effects of NRF2 activation in pancreatic cancer cells. ABC, ATP binding cassette; TGF-β1, transforming growth factor-β1.

Activation of NRF2 in pancreatic cancer has been identified in a cell line that is resistant to chemotherapeutic agents. The human pancreatic cancer cell line MIAPaCa-2 was exposed to low-dose gemcitabine for 6 months, and a gemcitabine-resistant cell line was established. This cell line showed increased intracellular ROS and NRF2 accumulation and elevated the expression of NRF2 target genes [69]. NRF2 knockdown by RNA interference sensitized pancreatic cancer cells to gemcitabine, suggesting that NRF2 activation is essential for acquiring resistance. Crosstalk between NRF2 and other cancer-promoting signals also contributes to the malignant phenotype. The inducer of EMT, transforming growth factor-β1 signaling, was attenuated by knockdown of NRF2 in pancreatic cancer cells [70]. PanIN lesions of surgically resected human pancreas tissue showed increased expression of nuclear NRF2 and decreased expression of E-cadherin, compared to normal pancreatic duct epithelium [70]. Accumulation of p62 also activated NRF2 in pancreatic cancer, leading to accelerated carcinogenesis. Pancreas-specific mutant *K-ras* expression and deletion of *IκB kinase α* promoted pancreatic cancer by increasing inflammation. The inflamed pancreatic parenchyma revealed p62 accumulation, and the deletion of p62 attenuated cancer progression [71]. The major oncogene *K-ras*, frequently mutated in pancreatic cancer, also activated Nrf2. Pancreas-specific expression of *K-ras*, together with other oncogenic *B-raf* mutations or *Myc* overexpression, resulted in the activation of Nrf2, which reduced intracellular ROS and increased cellular proliferation [72]. Introduction of *Nrf2-null* background into the KPC mouse, a pancreatic cancer model driven by pancreas-specific mutant *K-ras*/*p53* expression [73], delayed pancreatic cancer development via the attenuation of mRNA translation [74]. In this study, pancreatic organoids from KPC mice and *Nrf2-null* KPC mice were established, and *Nrf2-null* organoids showed vulnerability to AKT inhibition. Another study compared the development of precancerous lesions, pancreatic intraepithelial neoplasm (PanIN), and progression to invasive cancer between KPC mice and *Nrf2-null* KPC mice [75]. *Nrf2* deletion reduced both PanIN formation and progression to invasive cancer. In the *Nrf2-null* KPC mouse, PanIN lesions showed reduced expression of Nqo1 and increased 8-OHdG expression, a hallmark of increased oxidative stress. Cancer cell lines derived from *Nrf2-null* KPC mice showed lower expression of Nrf2 target genes, such as *ABC transporters*, *glutathione S-transferases*, and *UDP glucuronyl transferases*. Pancreatic cancer cell lines lacking Nrf2 were vulnerable to gemcitabine and the oxidative stress inducer, diethyl maleate (DEM). These studies highlighted the cancer-promoting role of NRF2 in pancreatic cancer (Figure 4).

However, the pancreas-specific constitutive activation of Nrf2 with mutant *K-ras* led to unexpected results. The addition of the pancreas-specific deletion of *Keap1* in KPC mice resulted in body weight loss and weakness, and most mice died within 2 months after birth [76]. In these mice, pancreatic tissues showed loss of acinar cells and islet cells, which were substituted by fibrous tissues. This phenomenon depended on mutant *K-ras* expression and *Keap1* deletion, and the Nrf2 target Nqo1 expression was elevated in the pancreas. This lethal phenotype, as well as progressive pancreatic atrophy, were rescued by the addition of *Nrf2-null* background or *Nrf2*^+/−^ background, suggesting that Nrf2 is associated with a specific threshold for the development of pancreatic atrophy. On the other hand, the same set of genetic alterations in the liver resulted in different outcomes. In the previous study, the liver-specific expression of mutant *K-ras* and deletion of *p53* in mice led to the development of cholangiocarcinoma [77]. The liver-specific mutant *K-ras* and *p53* expression with *Keap1* deletion accelerated cholangiocarcinoma formation. The addition of *Keap1* deletion caused an increase in Sox9/Nqo1 positive bile ducts, suggesting differentiation towards the ductal cell lineage [78]. In a lung cancer model, lung-specific *K-ras* expression and *Keap1* deletion worsened the survival of mice; however, attenuation of *Keap1* expression in immune cells improved survival [79]. This study suggested that Nrf2 activation in the cancer microenvironment suppresses progression. Collectively, NRF2 activation during carcinogenesis and roles of NRF2 are organ-, stage- and cellular context-specific [80].

## 8. Oxidative Stress and PSC Activation

**Figure 5:**
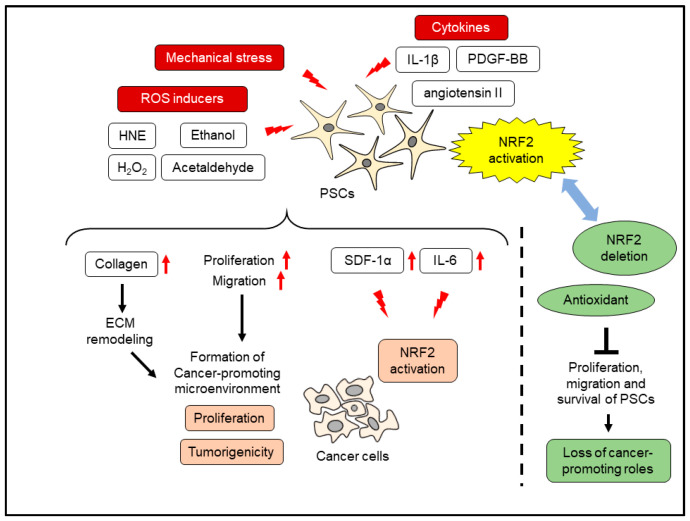
Effects of oxidative stress and NRF2 activation in PSCs. ECM, extracellular matrix; HNE, 4-hydroxy-2,3-nonenal; IL-1β, interleulin-1β; IL-6, interleukin-6; PDGF-BB, platelet-derived growth factor-BB; SDF-1α, stromal-derived factor-1 α.

In addition to hypoxia, oxidative stress activates PSCs. Stimulation of isolated PSCs with inducers of oxidative stress, such hydrogen peroxide, activated multiple signaling pathways [81]. This treatment increased collagen production, thereby promoting fibrosis. The key components of the ROS-producing enzyme NADPH oxidase were expressed in PSCs. DPI treatment attenuated platelet-derived growth factor-BB, interleulin-1β, and angiotensin II-induced ROS production, leading to the inhibition of PSC activation [82]. A wide variety of stimuli increase oxidative stress in PSCs, leading to their activation. Oxidative stress-inducing treatments such as ethanol, acetaldehyde, and high glucose activated PSCs [83,84], which were blocked by N-acetylcysteine treatment, suggesting that ROS plays a central role in PSC activation. Another free radical scavenger, edaravone, decreased inflammatory cytokine production and PSC activation in a dibutylin dichloride-induced chronic pancreatitis rat model [85]. PSCs also affected the oxidative stress response of cancer cells. PSC-derived interleukin-6 and stromal-derived factor-1 α activated NRF2 in pancreatic cancer cells, leading to increased proliferation and ROS detoxification [86]. These lines of evidence suggested that oxidative stress responses in PSCs substantially contribute to cancer progression. In a previous study, a global *Nrf2* knockout was introduced into KPC mice [75]. The *Nrf2-null* KPC mouse also lacked *Nrf2* in PSCs. There were less stromal cells surrounding PanINs in *Nrf2-null* KPC mice, suggesting attenuation of the cancer-promoting effects of PSCs. Indeed, PSCs isolated from *Nrf2-null* mice showed less proliferation, migration, and activation by serum stimulation [87]. *Nrf2-null* PSC-derived conditioned medium did not increase cancer cell proliferation in vivo. Furthermore, co-injection of *Nrf2-null* PSCs with cancer cells into the dorsal flank of immunodeficient mice failed to increase subcutaneous tumor size, compared to wild-type PSCs. The subcutaneous tumor derived from the *Nrf2-null* PSC co-injection contained fewer α-SMA-positive PSCs compared to the wild-type PSC co-injected tumor. Interestingly, even the co-injection of the *Nrf2-null* pancreatic cancer cell line and wild-type PSCs increased the size of subcutaneous tumors, suggesting that Nrf2 in PSCs plays a pivotal role in the tumor-promoting interaction between cancer cells and PSCs. The growth-promoting roles of conditioned medium from *Nrf2-null* PSCs were not recovered by N-acetylcysteine treatment, indicating that alteration of specific downstream targets of Nrf2 at basal condition, rather than under oxidative stress condition, is involved in these phenomena. These studies suggested cancer promoting roles of NRF2 in PSCs (Figure 5).

## 9. Application to Therapeutic Strategy

Because activation of stress response mechanisms plays a pivotal role in pancreatic cancer progression, these mechanisms might serve as novel therapeutic targets for pancreatic cancer. For hypoxia, inhibition of HIF-1 was used to overcome the malignant phenotype. The orally active HIF-1α translation inhibitor PX-478 sensitized pancreatic cancer cells to radiation, both in vitro and in vivo. This treatment caused acute tumor microvessel decompression and improved blood flow [88]. Hypoxic conditions have also been targeted using cytotoxins acting under hypoxic conditions. TX-2098, a hypoxic cytotoxin, decreased the viability of pancreatic cancer cells under hypoxic conditions and suppressed VEGF production. This agent also exhibited antitumor activity in a subcutaneous implantation model, suggesting in vivo efficacy [89]. Downstream molecules of HIF-1, such as CTGF, might also serve as therapeutic targets for pancreatic cancer. Pamrevlumab, a human monoclonal antibody that targets CTGF, enhanced the effects of neoadjuvant chemotherapy in pancreatic cancer [90]. Treatments with pamrevlumab increased the potential for surgical resection in patients with locally advanced pancreatic cancer. Treatment with this monoclonal antibody caused the cleavage of Ctgf in KPC mouse tumors, and reduced the expression of an antiapoptotic protein, the X-linked inhibitor of apoptosis protein [91]. Pancreatic cancer cells with methylthioadenosine phosphorylase deficiency revealed aberrant HIF-1 activation, and these cells were vulnerable to dual inhibition of glycolysis and de novo purine synthesis by 2-deoxy-d-glucose and l-alanosine, respectively [92]. Continuous activation of HIF-1 led to increased glycolysis and purine synthesis, creating an “Achilles’ heel” in cancer cells. These studies suggested that adaptation to certain environments can lead to dependence on specific metabolic pathways, thereby becoming targetable. On the other hand, several agents have been reported to suppress the activation of PSCs under hypoxic conditions. Administration of resveratrol, a polyphenolic compound, to a mutant *K-ras*/*p53* based pancreatic cancer mouse model repressed cancer progression and desmoplasia formation. In vitro experiments confirmed the inhibitory effects of resveratrol on interleukin-6, VEGF, and stromal-derived factor-1 α production in PSCs [93]. Recently, melatonin, produced by the pineal body, was shown to induce apoptosis in PSCs under hypoxic conditions. Melatonin treatment increased endoplasmic reticulum stress and apoptosis in PSCs in a dose-dependent manner [94]. 

Several NRF2 inhibitors have been reported to exhibit antitumor activity. For example, clobetasol propionate promoted NRF2 reduction by enhancing β-TrCP degradation. This treatment led to an increase in ROS and suppressed cellular proliferation in *KEAP1* mutated lung cancer cells [95]. Similarly, the small molecule inhibitor ML385, which interferes with DNA binding of the transcriptional complex containing NRF2 for the target gene, sensitized *KEAP1*-deficient lung cancer cells to carboplatin [96]. The compound NSC84167 could selectively induce apoptosis in NRF2-activated pancreatic cancer cells [97]. Using a reporter assay-based high-throughput assay, a recent study identified the plant alkaloid derivative, halofuginone, as a potent NRF2 inhibitor [98]. This agent acts as a potent inhibitor of protein synthesis, resulting in the depletion of proteins with short half-lives, such as NRF2. Halofuginone triggered an amino acid depletion reaction, represented by increased phosphorylation of eukaryotic translation initiation factor 2A and general control nonderepressible 2. This agent also attenuated the growth of NRF2-activated cancer cells, both in vitro and in vivo. In a study that tested the combined administration of halofuginone and gemcitabine in pancreatic cancer, halofuginone administration sensitized KPC mouse-derived pancreatic cancer cell lines to gemcitabine in vitro and in vivo, along with reduction of aldehyde dehydrogenase 3a1 (Aldh3a1) [99]. This effect was observed in halofuginone-treated KPC mouse pancreas, and the subcutaneous tumors of immunodeficient mice received KPC cell line implantation. The Nrf2 inducer DEM increased aldh3a1 expression in KPC mouse-derived pancreatic cancer cell lines, which was lost in *Nrf2-null* cell lines. Similarly, dexamethasone treatment decreased NRF2 expression in Panc-1 cells, which was attenuated by glucocorticoid receptor knockdown. Dexamethasone-sensitized Panc-1 cells to gemcitabine and 5-fluorouracil, and N-acetylcysteine blocked this effect [100]. In contrast, NRF2 activators also revealed therapeutic effects in experimental models of pancreatic cancer. Sulforaphane, the glucosinolate derivative from cruciferous vegetables, inhibited pancreatic cancer progression under high-glucose conditions. NRF2 activation inhibited cancer cell invasion in this context [101]. Another Nrf2 inducer, dimethyl fumarate (DMF), used in the treatment of multiple sclerosis, revealed antitumor effects in pancreatic cancer cells. DMF repressed mitochondrial respiration and glycolysis at the same time, leading to metabolic crisis and cancer cell death [102]. Further studies are warranted to clarify whether inhibition or activation of NRF2 become beneficial for pancreatic cancer treatment, according to biological contexts.

Metabolic reprogramming caused by stress responses may also be a therapeutic target. Lung cancer cells with constitutive NRF2 activation altered amino acid metabolism and purine nucleotide synthesis, yielding growth advantages [103]. NRF2-active *K-ras* mutant lung cancer cells showed increased sensitivity to CB-839, a glutaminase inhibitor [104]. Because pancreatic cancer cells frequently harbor mutant *K-ras*, a recent study assessed the relationship between NRF2 activation and CB-839 sensitivity in pancreatic cancer. Established murine pancreatic cancer cell lines from *Keap1-null*, *Nrf2*^+/−^ KPC mouse pancreatic cancer cells showed increased nuclear accumulation of Nrf2 [105]. These cell lines were more sensitive to CB-839 treatment than *Keap1-null* and *Nrf2-null* KPC mouse pancreatic cancer cell lines. Furthermore, combined treatment with DEM and CB-839 reduced the viability of KPC lines. This phenomenon was also observed in the *K-ras* mutant human pancreatic cancer cell lines, Panc-1 and MiaPaCa-2, but not in the *K-ras* wild-type BxPC3. The combination of NRF2 induction and certain interventions in the metabolic pathway may be a novel approach for pancreatic cancer. Inhibition of the NRF2 target also improved the efficacy of therapeutic intervention under hypoxic conditions. Inhibitors of heme oxygenase-1, zinc protoporphyrin, and tin protoporphyrin IX inhibited the proliferation of pancreatic cancer cells under hypoxia. Furthermore, treatment with these agents sensitized cancer cells to gemcitabine. Administration of zinc protoporphyrin and gemcitabine to immunodeficient mice bearing orthotopic implantation of pancreatic cancer reduced tumor weight and metastasis [106]. Disrupting the NRF2-HIF-interaction might be useful for overcoming the hypoxia-induced resistance of pancreatic cancer cells [107]. Pancreatic cancer cells undergo tidal changes in the tumor microenvironment, especially hypoxia and oxidative stress. Further studies are needed to clarify the crosstalk between hypoxia responses and oxidative stress responses.

## 10. Conclusions

In this review, we have summarized the current knowledge on stress response mechanisms and pancreatic cancer progression. Activation of these stress responses is indispensable for the survival of cancer cells and the acquisition of a malignant phenotype. In addition, stress responses in PSCs also play a pivotal role in establishing a cancer-promoting microenvironment. The use of HIF-1 or NRF2 inhibitors revealed efficacy in vitro and in vivo, which requires further validation in clinical settings. Recently, the concept referred to as “synthetic lethality” is emerging in the cancer research field [108]. Dependence to stress response mechanisms in cancer cells or PSCs could be an ideal target for this therapy concept. Sensitization to glutaminase inhibition by NRF2 activation is a good example [105]. Dissection of these complex mechanisms is essential for the development of effective therapies for pancreatic cancer.

## References

[1] Park W., Chawla A., O’Reilly E.M. (2021). Pancreatic cancer: A review. JAMA.

[2] Okusaka T., Furuse J. (2020). Recent advances in chemotherapy for pancreatic cancer: Evidence from Japan and recommendations in guidelines. J. Gastroenterol..

[3] Yachida S., Jones S., Bozic I., Antal T., Leary R., Fu B., Kamiyama M., Hruban R.H., Eshleman J.R., Nowak M.A. (2010). Distant metastasis occurs late during the genetic evolution of pancreatic cancer. Nature.

[4] Samulitis B.K., Pond K.W., Pond E., Cress A.E., Patel H., Wisner L., Patel C., Dorr R.T., Landowski T.H. (2015). Gemcitabine resistant pancreatic cancer cell lines acquire an invasive phenotype with collateral hypersensitivity to histone deacetylase inhibitors. Cancer Biol. Ther..

[5] Masamune A., Shimosegawa T. (2015). Pancreatic stellate cells: A dynamic player of the intercellular communication in pancreatic cancer. Clin. Res. Hepatol. Gastroenterol..

[6] Erkan M., Adler G., Apte M.V., Bachem M.G., Buchholz M., Detlefsen S., Esposito I., Friess H., Gress T.M., Habisch H.J. (2012). StellaTUM: Current consensus and discussion on pancreatic stellate cell research. Gut.

[7] Kikuta K., Masamune A., Watanabe T., Ariga H., Itoh H., Hamada S., Satoh K., Egawa S., Unno M., Shimosegawa T. (2010). Pancreatic stellate cells promote epithelial-mesenchymal transition in pancreatic cancer cells. Biochem. Biophys. Res. Commun..

[8] Hamada S., Masamune A., Takikawa T., Suzuki N., Kikuta K., Hirota M., Hamada H., Kobune M., Satoh K., Shimosegawa T. (2012). Pancreatic stellate cells enhance stem cell-like phenotypes in pancreatic cancer cells. Biochem. Biophys. Res. Commun..

[9] Hamada S., Masamune A., Yoshida N., Takikawa T., Shimosegawa T. (2016). IL-6/STAT3 plays a regulatory role in the interaction between pancreatic stellate cells and cancer cells. Dig. Dis. Sci..

[10] Olive K.P., Jacobetz M.A., Davidson C.J., Gopinathan A., McIntyre D., Honess D., Madhu B., Goldgraben M.A., Caldwell M.E., Allard D. (2009). Inhibition of Hedgehog signaling enhances delivery of chemotherapy in a mouse model of pancreatic cancer. Science.

[11] Ozdemir B.C., Pentcheva-Hoang T., Carstens J.L., Zheng X., Wu C.C., Simpson T.R., Laklai H., Sugimoto H., Kahlert C., Novitskiy S.V. (2014). Depletion of carcinoma-associated fibroblasts and fibrosis induces immunosuppression and accelerates pancreas cancer with reduced survival. Cancer Cell.

[12] Mizutani Y., Kobayashi H., Iida T., Asai N., Masamune A., Hara A., Esaki N., Ushida K., Mii S., Shiraki Y. (2019). Meflin-positive cancer-associated fibroblasts inhibit pancreatic carcinogenesis. Cancer Res..

[13] Guillaumond F., Iovanna J.L., Vasseur S. (2014). Pancreatic tumor cell metabolism: Focus on glycolysis and its connected metabolic pathways. Arch. Biochem. Biophys..

[14] Carvalho T.M.A., Di Molfetta D., Greco M.R., Koltai T., Alfarouk K.O., Reshkin S.J., Cardone R.A. (2021). Tumor microenvironment features and chemoresistance in pancreatic ductal adenocarcinoma: Insights into targeting physicochemical barriers and metabolism as therapeutic approaches. Cancers.

[15] Huang X., He C., Hua X., Kan A., Mao Y., Sun S., Duan F., Wang J., Huang P., Li S. (2020). Oxidative stress induces monocyte-to-myofibroblast transdifferentiation through p38 in pancreatic ductal adenocarcinoma. Clin. Transl. Med..

[16] Corzo C.A., Cotter M.J., Cheng P., Cheng F., Kusmartsev S., Sotomayor E., Padhya T., McCaffrey T.V., McCaffrey J.C., Gabrilovich D.I. (2009). Mechanism regulating reactive oxygen species in tumor-induced myeloid-derived suppressor cells. J. Immunol..

[17] Seong J.B., Kim B., Kim S., Kim M.H., Park Y.H., Lee Y., Lee H.J., Hong C.W., Lee D.S. (2021). Macrophage peroxiredoxin 5 deficiency promotes lung cancer progression via ROS-dependent M2-like polarization. Free Radic. Biol. Med..

[18] Edderkaoui M., Hong P., Vaquero E., Lee J., Fischer L., Friess H., Buchler M., Lerch M., Pandol S.J., Gukovskaya A. (2005). Extracellular matrix stimulates reactive oxygen species production and increases pancreatic cancer cell survival through 5-lipoxygenase and NADPH oxidase. Am. J. Physiol. Gastrointest. Liver Physiol..

[19] Wang Y., Qi H., Liu Y., Duan C., Liu X., Xia T., Chen D., Piao H.L., Liu H.X. (2021). The double-edged roles of ROS in cancer prevention and therapy. Theranostics.

[20] Zhang L., Li J., Zong L., Chen X., Chen K., Jiang Z., Nan L., Li X., Li W., Shan T. (2016). Reactive oxygen species and targeted therapy for pancreatic cancer. Oxid. Med. Cell. Longev..

[21] Gervasoni J.E., Hindenburg A.A., Vezeridis M.P., Schulze S., Wanebo H.J., Mehta S. (2004). An effective in vitro antitumor response against human pancreatic carcinoma with paclitaxel and daunorubicin by induction of both necrosis and apoptosis. Anticancer Res..

[22] Infantino V., Santarsiero A., Convertini P., Todisco S., Iacobazzi V. (2021). Cancer cell metabolism in hypoxia: Role of HIF-1 as key regulator and therapeutic target. Int. J. Mol. Sci..

[23] Baird L., Yamamoto M. (2020). The molecular mechanisms regulating the KEAP1-NRF2 pathway. Mol. Cell Biol..

[24] Koong A.C., Mehta V.K., Le Q.T., Fisher G.A., Terris D.J., Brown J.M., Bastidas A.J., Vierra M. (2000). Pancreatic tumors show high levels of hypoxia. Int. J. Radiat. Oncol. Biol. Phys..

[25] Morfoisse F., Renaud E., Hantelys F., Prats A.C., Garmy-Susini B. (2015). Role of hypoxia and vascular endothelial growth factors in lymphangiogenesis. Mol. Cell Oncol..

[26] Nakazawa M.S., Keith B., Simon M.C. (2016). Oxygen availability and metabolic adaptations. Nat. Rev. Cancer.

[27] Xie H., Simon M.C. (2017). Oxygen availability and metabolic reprogramming in cancer. J. Biol. Chem..

[28] Morotti M., Bridges E., Valli A., Choudhry H., Sheldon H., Wigfield S., Gray N., Zois C.E., Grimm F., Jones D. (2019). Hypoxia-induced switch in SNAT2/SLC38A2 regulation generates endocrine resistance in breast cancer. Proc. Natl. Acad. Sci. USA.

[29] Latif F., Tory K., Gnarra J., Yao M., Duh F.M., Orcutt M.L., Stackhouse T., Kuzmin I., Modi W., Geil L. (1993). Identification of the von Hippel-Lindau disease tumor suppressor gene. Science.

[30] Natsuizaka M., Ozasa M., Darmanin S., Miyamoto M., Kondo S., Kamada S., Shindoh M., Higashino F., Suhara W., Koide H. (2007). Synergistic up-regulation of Hexokinase-2, glucose transporters and angiogenic factors in pancreatic cancer cells by glucose deprivation and hypoxia. Exp. Cell Res..

[31] Kitajima Y., Ide T., Ohtsuka T., Miyazaki K. (2008). Induction of hepatocyte growth factor activator gene expression under hypoxia activates the hepatocyte growth factor/c-Met system via hypoxia inducible factor-1 in pancreatic cancer. Cancer Sci..

[32] Buchler P., Reber H.A., Tomlinson J.S., Hankinson O., Kallifatidis G., Friess H., Herr I., Hines O.J. (2009). Transcriptional regulation of urokinase-type plasminogen activator receptor by hypoxia-inducible factor 1 is crucial for invasion of pancreatic and liver cancer. Neoplasia.

[33] Cao X.P., Cao Y., Li W.J., Zhang H.H., Zhu Z.M. (2019). P4HA1/HIF1alpha feedback loop drives the glycolytic and malignant phenotypes of pancreatic cancer. Biochem. Biophys. Res. Commun..

[34] Papandreou I., Cairns R.A., Fontana L., Lim A.L., Denko N.C. (2006). HIF-1 mediates adaptation to hypoxia by actively downregulating mitochondrial oxygen consumption. Cell Metab..

[35] Golias T., Papandreou I., Sun R., Kumar B., Brown N.V., Swanson B.J., Pai R., Jaitin D., Le Q.T., Teknos T.N. (2016). Hypoxic repression of pyruvate dehydrogenase activity is necessary for metabolic reprogramming and growth of model tumours. Sci. Rep..

[36] Chen S., Chen J.Z., Zhang J.Q., Chen H.X., Yan M.L., Huang L., Tian Y.F., Chen Y.L., Wang Y.D. (2016). Hypoxia induces TWIST-activated epithelial-mesenchymal transition and proliferation of pancreatic cancer cells in vitro and in nude mice. Cancer Lett..

[37] Higgins D.F., Biju M.P., Akai Y., Wutz A., Johnson R.S., Haase V.H. (2004). Hypoxic induction of Ctgf is directly mediated by Hif-1. Am. J. Physiol. Renal Physiol..

[38] Hong K.H., Yoo S.A., Kang S.S., Choi J.J., Kim W.U., Cho C.S. (2006). Hypoxia induces expression of connective tissue growth factor in scleroderma skin fibroblasts. Clin. Exp. Immunol..

[39] Bennewith K.L., Huang X., Ham C.M., Graves E.E., Erler J.T., Kambham N., Feazell J., Yang G.P., Koong A., Giaccia A.J. (2009). The role of tumor cell-derived connective tissue growth factor (CTGF/CCN2) in pancreatic tumor growth. Cancer Res..

[40] Maity G., Ghosh A., Gupta V., Haque I., Sarkar S., Das A., Dhar K., Bhavanasi S., Gunewardena S.S., Von Hoff D.D. (2019). CYR61/CCN1 regulates dCK and CTGF and causes gemcitabine-resistant phenotype in pancreatic ductal adenocarcinoma. Mol. Cancer Ther..

[41] Jin X., Dai L., Ma Y., Wang J., Liu Z. (2020). Implications of HIF-1alpha in the tumorigenesis and progression of pancreatic cancer. Cancer Cell Int..

[42] Mace T.A., Collins A.L., Wojcik S.E., Croce C.M., Lesinski G.B., Bloomston M. (2013). Hypoxia induces the overexpression of microRNA-21 in pancreatic cancer cells. J. Surg. Res..

[43] Greco S., Martelli F. (2014). MicroRNAs in hypoxia response. Antioxid. Redox Signal..

[44] Li B., Cao Y., Sun M., Feng H. (2021). Expression, regulation, and function of exosome-derived miRNAs in cancer progression and therapy. FASEB J..

[45] Greither T., Grochola L.F., Udelnow A., Lautenschlager C., Wurl P., Taubert H. (2010). Elevated expression of microRNAs 155, 203, 210 and 222 in pancreatic tumors is associated with poorer survival. Int. J. Cancer.

[46] Takikawa T., Masamune A., Hamada S., Nakano E., Yoshida N., Shimosegawa T. (2013). miR-210 regulates the interaction between pancreatic cancer cells and stellate cells. Biochem. Biophys. Res. Commun..

[47] Giovannetti E., Funel N., Peters G.J., Del Chiaro M., Erozenci L.A., Vasile E., Leon L.G., Pollina L.E., Groen A., Falcone A. (2010). MicroRNA-21 in pancreatic cancer: Correlation with clinical outcome and pharmacologic aspects underlying its role in the modulation of gemcitabine activity. Cancer Res..

[48] Luo G., Xia X., Wang X., Zhang K., Cao J., Jiang T., Zhao Q., Qiu Z. (2018). miR-301a plays a pivotal role in hypoxia-induced gemcitabine resistance in pancreatic cancer. Exp. Cell Res..

[49] Wang X., Luo G., Zhang K., Cao J., Huang C., Jiang T., Liu B., Su L., Qiu Z. (2018). Hypoxic tumor-derived exosomal miR-301a mediates M2 macrophage polarization via PTEN/PI3Kgamma to promote pancreatic cancer metastasis. Cancer Res..

[50] Masamune A., Kikuta K., Watanabe T., Satoh K., Hirota M., Shimosegawa T. (2008). Hypoxia stimulates pancreatic stellate cells to induce fibrosis and angiogenesis in pancreatic cancer. Am. J. Physiol. Gastrointest. Liver Physiol..

[51] Erkan M., Reiser-Erkan C., Michalski C.W., Deucker S., Sauliunaite D., Streit S., Esposito I., Friess H., Kleeff J. (2009). Cancer-stellate cell interactions perpetuate the hypoxia-fibrosis cycle in pancreatic ductal adenocarcinoma. Neoplasia.

[52] Eguchi D., Ikenaga N., Ohuchida K., Kozono S., Cui L., Fujiwara K., Fujino M., Ohtsuka T., Mizumoto K., Tanaka M. (2013). Hypoxia enhances the interaction between pancreatic stellate cells and cancer cells via increased secretion of connective tissue growth factor. J. Surg. Res..

[53] Sada M., Ohuchida K., Horioka K., Okumura T., Moriyama T., Miyasaka Y., Ohtsuka T., Mizumoto K., Oda Y., Nakamura M. (2016). Hypoxic stellate cells of pancreatic cancer stroma regulate extracellular matrix fiber organization and cancer cell motility. Cancer Lett..

[54] Kikuta K., Masamune A., Hamada S., Takikawa T., Nakano E., Shimosegawa T. (2013). Pancreatic stellate cells reduce insulin expression and induce apoptosis in pancreatic beta-cells. Biochem. Biophys. Res. Commun..

[55] Kim J.J., Lee E., Ryu G.R., Ko S.H., Ahn Y.B., Song K.H. (2020). Hypoxia increases beta-cell death by activating pancreatic stellate cells within the islet. Diabetes Metab. J..

[56] Li X., Truty M.A., Kang Y., Chopin-Laly X., Zhang R., Roife D., Chatterjee D., Lin E., Thomas R.M., Wang H. (2014). Extracellular lumican inhibits pancreatic cancer cell growth and is associated with prolonged survival after surgery. Clin. Cancer Res..

[57] Li X., Lee Y., Kang Y., Dai B., Perez M.R., Pratt M., Koay E.J., Kim M., Brekken R.A., Fleming J.B. (2019). Hypoxia-induced autophagy of stellate cells inhibits expression and secretion of lumican into microenvironment of pancreatic ductal adenocarcinoma. Cell Death Differ..

[58] Li N., Li Y., Li Z., Huang C., Yang Y., Lang M., Cao J., Jiang W., Xu Y., Dong J. (2016). Hypoxia inducible factor 1 (HIF-1) recruits macrophage to activate pancreatic stellate cells in pancreatic ductal adenocarcinoma. Int. J. Mol. Sci..

[59] Cao W., Zeng Z., He Z., Lei S. (2021). Hypoxic pancreatic stellate cell-derived exosomal mirnas promote proliferation and invasion of pancreatic cancer through the PTEN/AKT pathway. Aging.

[60] Taguchi K., Yamamoto M. (2020). The KEAP1-NRF2 system as a molecular target of cancer treatment. Cancers.

[61] Yamamoto M., Kensler T.W., Motohashi H. (2018). The KEAP1-NRF2 system: A thiol-based sensor-effector apparatus for maintaining redox homeostasis. Physiol. Rev..

[62] Taguchi K., Yamamoto M. (2017). The KEAP1-NRF2 system in cancer. Front. Oncol..

[63] Frank R., Scheffler M., Merkelbach-Bruse S., Ihle M.A., Kron A., Rauer M., Ueckeroth F., Konig K., Michels S., Fischer R. (2018). Clinical and pathological characteristics of KEAP1- and NFE2L2-mutated non-small cell lung carcinoma (NSCLC). Clin. Cancer Res..

[64] Kerins M.J., Ooi A. (2018). A catalogue of somatic NRF2 gain-of-function mutations in cancer. Sci. Rep..

[65] Shibata T., Kokubu A., Saito S., Narisawa-Saito M., Sasaki H., Aoyagi K., Yoshimatsu Y., Tachimori Y., Kushima R., Kiyono T. (2011). NRF2 mutation confers malignant potential and resistance to chemoradiation therapy in advanced esophageal squamous cancer. Neoplasia.

[66] Umemura A., He F., Taniguchi K., Nakagawa H., Yamachika S., Font-Burgada J., Zhong Z., Subramaniam S., Raghunandan S., Duran A. (2016). p62, upregulated during preneoplasia, induces hepatocellular carcinogenesis by maintaining survival of stressed HCC-initiating cells. Cancer Cell.

[67] Saito T., Ichimura Y., Taguchi K., Suzuki T., Mizushima T., Takagi K., Hirose Y., Nagahashi M., Iso T., Fukutomi T. (2016). p62/Sqstm1 promotes malignancy of HCV-positive hepatocellular carcinoma through Nrf2-dependent metabolic reprogramming. Nat. Commun..

[68] Purohit V., Wang L., Yang H., Li J., Ney G.M., Gumkowski E.R., Vaidya A.J., Wang A., Bhardwaj A., Zhao E. (2021). ATDC binds to KEAP1 to drive NRF2-mediated tumorigenesis and chemoresistance in pancreatic cancer. Genes Dev..

[69] Ju H.Q., Gocho T., Aguilar M., Wu M., Zhuang Z.N., Fu J., Yanaga K., Huang P., Chiao P.J. (2015). Mechanisms of overcoming intrinsic resistance to gemcitabine in pancreatic ductal adenocarcinoma through the redox modulation. Mol. Cancer Ther..

[70] Arfmann-Knubel S., Struck B., Genrich G., Helm O., Sipos B., Sebens S., Schafer H. (2015). The crosstalk between Nrf2 and TGF-beta1 in the epithelial-mesenchymal transition of pancreatic duct epithelial cells. PLoS ONE.

[71] Todoric J., Antonucci L., Di Caro G., Li N., Wu X., Lytle N.K., Dhar D., Banerjee S., Fagman J.B., Browne C.D. (2017). Stress-activated NRF2-MDM2 cascade controls neoplastic progression in pancreas. Cancer Cell.

[72] DeNicola G.M., Karreth F.A., Humpton T.J., Gopinathan A., Wei C., Frese K., Mangal D., Yu K.H., Yeo C.J., Calhoun E.S. (2011). Oncogene-induced Nrf2 transcription promotes ROS detoxification and tumorigenesis. Nature.

[73] Hingorani S.R., Wang L., Multani A.S., Combs C., Deramaudt T.B., Hruban R.H., Rustgi A.K., Chang S., Tuveson D.A. (2005). Trp53R172H and KrasG12D cooperate to promote chromosomal instability and widely metastatic pancreatic ductal adenocarcinoma in mice. Cancer Cell.

[74] Chio I.I.C., Jafarnejad S.M., Ponz-Sarvise M., Park Y., Rivera K., Palm W., Wilson J., Sangar V., Hao Y., Ohlund D. (2016). NRF2 promotes tumor maintenance by modulating mRNA translation in pancreatic cancer. Cell.

[75] Hamada S., Taguchi K., Masamune A., Yamamoto M., Shimosegawa T. (2017). Nrf2 promotes mutant K-ras/p53-driven pancreatic carcinogenesis. Carcinogenesis.

[76] Hamada S., Shimosegawa T., Taguchi K., Nabeshima T., Yamamoto M., Masamune A. (2018). Simultaneous K-ras activation and Keap1 deletion cause atrophy of pancreatic parenchyma. Am. J. Physiol. Gastrointest. Liver Physiol..

[77] O’Dell M.R., Huang J.L., Whitney-Miller C.L., Deshpande V., Rothberg P., Grose V., Rossi R.M., Zhu A.X., Land H., Bardeesy N. (2012). Kras(G12D) and p53 mutation cause primary intrahepatic cholangiocarcinoma. Cancer Res..

[78] Nabeshima T., Hamada S., Taguchi K., Tanaka Y., Matsumoto R., Yamamoto M., Masamune A. (2020). Keap1 deletion accelerates mutant K-ras/p53-driven cholangiocarcinoma. Am. J. Physiol. Gastrointest. Liver Physiol..

[79] Hayashi M., Kuga A., Suzuki M., Panda H., Kitamura H., Motohashi H., Yamamoto M. (2020). Microenvironmental activation of Nrf2 restricts the progression of Nrf2-activated malignant tumors. Cancer Res..

[80] Qin J.J., Cheng X.D., Zhang J., Zhang W.D. (2019). Dual roles and therapeutic potential of Keap1-Nrf2 pathway in pancreatic cancer: A systematic review. Cell Commun. Signal..

[81] Yan B., Cheng L., Jiang Z., Chen K., Zhou C., Sun L., Cao J., Qian W., Li J., Shan T. (2018). Resveratrol inhibits ROS-promoted activation and glycolysis of pancreatic stellate cells via suppression of miR-21. Oxid. Med. Cell Longev..

[82] Masamune A., Watanabe T., Kikuta K., Satoh K., Shimosegawa T. (2008). NADPH oxidase plays a crucial role in the activation of pancreatic stellate cells. Am. J. Physiol. Gastrointest. Liver Physiol..

[83] Masamune A., Kikuta K., Satoh M., Satoh A., Shimosegawa T. (2002). Alcohol activates activator protein-1 and mitogen-activated protein kinases in rat pancreatic stellate cells. J. Pharmacol. Exp. Ther..

[84] Ryu G.R., Lee E., Chun H.J., Yoon K.H., Ko S.H., Ahn Y.B., Song K.H. (2013). Oxidative stress plays a role in high glucose-induced activation of pancreatic stellate cells. Biochem. Biophys. Res. Commun..

[85] Zhou C.H., Lin L., Zhu X.Y., Wen T., Hu D.M., Dong Y., Li L.Y., Wang S.F. (2013). Protective effects of edaravone on experimental chronic pancreatitis induced by dibutyltin dichloride in rats. Pancreatology.

[86] Wu Y.S., Looi C.Y., Subramaniam K.S., Masamune A., Chung I. (2016). Soluble factors from stellate cells induce pancreatic cancer cell proliferation via Nrf2-activated metabolic reprogramming and ROS detoxification. Oncotarget.

[87] Tanaka Y., Hamada S., Matsumoto R., Taguchi K., Yamamoto M., Masamune A. (2021). Nrf2 expression in pancreatic stellate cells promotes progression of cancer. Am. J. Physiol. Gastrointest. Liver Physiol..

[88] Schwartz D.L., Bankson J.A., Lemos R., Lai S.Y., Thittai A.K., He Y., Hostetter G., Demeure M.J., Von Hoff D.D., Powis G. (2010). Radiosensitization and stromal imaging response correlates for the HIF-1 inhibitor PX-478 given with or without chemotherapy in pancreatic cancer. Mol. Cancer Ther..

[89] Miyake K., Nishioka M., Imura S., Batmunkh E., Uto Y., Nagasawa H., Hori H., Shimada M. (2012). The novel hypoxic cytotoxin, TX-2098 has antitumor effect in pancreatic cancer; possible mechanism through inhibiting VEGF and hypoxia inducible factor-1alpha targeted gene expression. Exp. Cell Res..

[90] Picozzi V., Alseidi A., Winter J., Pishvaian M., Mody K., Glaspy J., Larson T., Matrana M., Carney M., Porter S. (2020). Gemcitabine/nab-paclitaxel with pamrevlumab: A novel drug combination and trial design for the treatment of locally advanced pancreatic cancer. ESMO Open.

[91] Neesse A., Frese K.K., Bapiro T.E., Nakagawa T., Sternlicht M.D., Seeley T.W., Pilarsky C., Jodrell D.I., Spong S.M., Tuveson D.A. (2013). CTGF antagonism with mAb FG-3019 enhances chemotherapy response without increasing drug delivery in murine ductal pancreas cancer. Proc. Natl. Acad. Sci. USA.

[92] Hu Q., Qin Y., Ji S., Shi X., Dai W., Fan G., Li S., Xu W., Liu W., Liu M. (2021). MTAP deficiency-induced metabolic reprogramming creates a vulnerability to cotargeting de novo purine synthesis and glycolysis in pancreatic cancer. Cancer Res..

[93] Xiao Y., Qin T., Sun L., Qian W., Li J., Duan W., Lei J., Wang Z., Ma J., Li X. (2020). Resveratrol ameliorates the malignant progression of pancreatic cancer by inhibiting hypoxia-induced pancreatic stellate cell activation. Cell Transplant..

[94] Estaras M., Gonzalez-Portillo M.R., Fernandez-Bermejo M., Mateos J.M., Vara D., Blanco-Fernandez G., Lopez-Guerra D., Roncero V., Salido G.M., Gonzalez A. (2021). Melatonin induces apoptosis and modulates cyclin expression and MAPK phosphorylation in pancreatic stellate cells subjected to hypoxia. Int. J. Mol. Sci..

[95] Choi E.J., Jung B.J., Lee S.H., Yoo H.S., Shin E.A., Ko H.J., Chang S., Kim S.Y., Jeon S.M. (2017). A clinical drug library screen identifies clobetasol propionate as an NRF2 inhibitor with potential therapeutic efficacy in KEAP1 mutant lung cancer. Oncogene.

[96] Singh A., Venkannagari S., Oh K.H., Zhang Y.Q., Rohde J.M., Liu L., Nimmagadda S., Sudini K., Brimacombe K.R., Gajghate S. (2016). Small molecule inhibitor of NRF2 selectively intervenes therapeutic resistance in KEAP1-deficient NSCLC tumors. ACS Chem. Biol..

[97] Dai B., Augustine J.J., Kang Y., Roife D., Li X., Deng J., Tan L., Rusling L.A., Weinstein J.N., Lorenzi P.L. (2021). Compound NSC84167 selectively targets NRF2-activated pancreatic cancer by inhibiting asparagine synthesis pathway. Cell Death Dis..

[98] Tsuchida K., Tsujita T., Hayashi M., Ojima A., Keleku-Lukwete N., Katsuoka F., Otsuki A., Kikuchi H., Oshima Y., Suzuki M. (2017). Halofuginone enhances the chemo-sensitivity of cancer cells by suppressing NRF2 accumulation. Free Radic. Biol. Med..

[99] Matsumoto R., Hamada S., Tanaka Y., Taguchi K., Yamamoto M., Masamune A. (2021). Nuclear factor erythroid 2-related factor 2 depletion sensitizes pancreatic cancer cells to gemcitabine via aldehyde dehydrogenase 3a1 repression. J. Pharmacol. Exp. Ther..

[100] Suzuki S., Yamamoto M., Sanomachi T., Togashi K., Sugai A., Seino S., Yoshioka T., Okada M., Kitanaka C. (2021). Dexamethasone sensitizes cancer stem cells to gemcitabine and 5-Fluorouracil by increasing reactive oxygen species production through NRF2 reduction. Life.

[101] Chen X., Jiang Z., Zhou C., Chen K., Li X., Wang Z., Wu Z., Ma J., Ma Q., Duan W. (2018). Activation of Nrf2 by sulforaphane inhibits high glucose-induced progression of pancreatic cancer via AMPK dependent signaling. Cell. Physiol. Biochem..

[102] Chen K., Wu S., Ye S., Huang H., Zhou Y., Zhou H., Wu S., Mao Y., Shangguan F., Lan L. (2021). Dimethyl fumarate induces metabolic crisie to suppress pancreatic carcinoma. Front. Pharmacol..

[103] Mitsuishi Y., Taguchi K., Kawatani Y., Shibata T., Nukiwa T., Aburatani H., Yamamoto M., Motohashi H. (2012). Nrf2 redirects glucose and glutamine into anabolic pathways in metabolic reprogramming. Cancer Cell.

[104] Galan-Cobo A., Sitthideatphaiboon P., Qu X., Poteete A., Pisegna M.A., Tong P., Chen P.H., Boroughs L.K., Rodriguez M.L.M., Zhang W. (2019). LKB1 and KEAP1/NRF2 pathways cooperatively promote metabolic reprogramming with enhanced glutamine dependence in KRAS-mutant lung adenocarcinoma. Cancer Res..

[105] Hamada S., Matsumoto R., Tanaka Y., Taguchi K., Yamamoto M., Masamune A. (2021). Nrf2 activation sensitizes K-Ras mutant pancreatic cancer cells to glutaminase inhibition. Int. J. Mol. Sci..

[106] Abdalla M.Y., Ahmad I.M., Rachagani S., Banerjee K., Thompson C.M., Maurer H.C., Olive K.P., Bailey K.L., Britigan B.E., Kumar S. (2019). Enhancing responsiveness of pancreatic cancer cells to gemcitabine treatment under hypoxia by heme oxygenase-1 inhibition. Transl. Res..

[107] Kuper A., Baumann J., Gopelt K., Baumann M., Sanger C., Metzen E., Kranz P., Brockmeier U. (2021). Overcoming hypoxia-induced resistance of pancreatic and lung tumor cells by disrupting the PERK-NRF2-HIF-axis. Cell Death Dis..

[108] Setton J., Zinda M., Riaz N., Durocher D., Zimmermann M., Koehler M., Reis-Filho J.S., Powell S.N. (2021). Synthetic lethality in cancer therapeutics: The next generation. Cancer Discov..

